# Dynamic Echocardiographic Assessments Reveal Septal E/e’ Ratio as Independent Predictor of Intradialytic Hypotension in Maintenance for Hemodialysis Patients with Preserved Ejection Fraction

**DOI:** 10.3390/diagnostics11122266

**Published:** 2021-12-03

**Authors:** Chun-Yu Chen, Ning-I Yang, Chin-Chan Lee, Ming-Jui Hung, Wen-Jin Cherng, Heng-Jung Hsu, Chiao-Yin Sun, I-Wen Wu

**Affiliations:** 1 Department of Nephrology, Chang Gung Memorial Hospital, Keelung 204, Taiwan; shone@cgmh.org.tw (C.-Y.C.); leefang@cgmh.org.tw (C.-C.L.); r5267@cgmh.org.tw (H.-J.H.); sun3970@cgmh.org.tw (C.-Y.S.); 2 College of Medicine, Chang Gung University, Taoyuan 333, Taiwan; hmj1447@cgmh.org.tw (M.-J.H.); cwenjin@cgmh.org.tw (W.-J.C.); 3 Department of Cardiology, Chang Gung Memorial Hospital, Keelung 204, Taiwan; r3675@cgmh.org.tw; 4 Department of Cardiology, Chang Gung Memorial Hospital, Linkou Branch, Taoyuan 333, Taiwan

**Keywords:** two-dimensional echocardiography, intradialytic hypotension, left ventricular diastolic dysfunction, biomarker, kidney

## Abstract

Background: Intradialytic hypotension (IDH) is a frequent and grave complication of hemodialysis (HD). However, the dynamic hemodynamic changes and cardiac performances during each dialytic session have been rarely explored in patients having IDH. Methods: Seventy-six HD patients (IDH = 40, controls = 36) were enrolled. Echocardiography examinations were performed in all patients at the pre-HD, during-HD and post-HD phases of a single HD session. A two-way analysis of variance was applied to compare differences of echocardiographic parameters between IDH and controls over time. The risk association was estimated by using a logistic regression analysis. Results: The IDH patients had a higher ejection fraction during HD followed by a greater reduction at the post-HD phase than the controls. Significant decreases in septal ratios of transmitral flow velocity to annular velocity (E/e’) over times were detected between IDH patients and controls after adjusting for gender, age and ultrafiltration (*p* = 0.016). A lower septal E/e’ ratio was independently associated with IDH (OR = 0.040; 95% CI = 0.003–0.606; *p* = 0.02). In contrast, significant systolic and diastolic dysfunctions over time were found in diabetic IDH compared to non-diabetic counterparts. Conclusion: The septal E/e’ ratio was a significant predictor for IDH.

## 1. Introduction

Hemodynamic instability represents an important clinical challenge in the management of hemodialysis (HD) patients. Intradialytic hypotension (IDH) often develops in HD patients having a high interdialytic body weight gain and it is associated with cardiovascular morbidities and mortality, as well as many acute and chronic complications [1,2]. Acute complications related to IDH include myocardial ischemia, cerebrovascular events and intestine ischemia. On the other hand, the chronic occurrence of IDH can lead to volume overloading, vascular access dysfunction and dialysis inadequacy because of premature discontinuation of the dialysis session. Possible pathophysiological mechanisms for development of IDH include inadequate cardiovascular reaction to compensate the decline of intravascular fluid volume from rapid ultrafiltration over a short time of several hours, deficient venous return system, defective arterial resistance and autonomic system dysfunction [3,4].

Echocardiography is the principal imaging modality used for the clinical evaluation of left ventricular (LV) systolic and diastolic function. The echocardiographic diagnosis of LV diastolic dysfunction with preserved ejection fraction is mainly based on the prolongation of deceleration time (DT), a reflection of slow and prolonged LV pressure decay that achieves only a modest decline in LV minimal pressure; increased septal ratios of transmitral flow velocity to annular velocity (E/e’), predicting an increased left atrium pressure (LAP); and increased overall mitral inflow peak E to peak A velocity ratios (E/A), suggesting an increased LAP [5,6]. The increased left atrium maximum volume index and LV end-diastolic volume (EDV) are often used as an assessment of diastolic function [7]. 

Most studies have reported LV systolic dysfunction and inadequate heart compensation leading to IDH [8,9,10], and repeated IDH would contribute to cardiac remodeling, including LV hypertrophy and systolic heart failure [11]. However, diastolic dysfunction, characterized by impaired ventricular relaxation, secondary to LV hypertrophy, can also develop in the early stage of chronic kidney disease (CKD) [3,4]. The serial hemodynamic changes and cardiac performance during each dialytic session have rarely been explored in IDH patients with a preserved ejection fraction who have no obvious symptoms of heart failure in interdialytic duration. In this case–control observational study, we conducted serial measurements of two-dimensional (2D) echocardiography to assess LV systolic and diastolic function and non-invasively measured LV filling pressures as assessed by septal annular velocity, before, during and after a single session of dialysis therapy in chronic HD patients with and without IDH.

## 2. Materials and Methods

### 2.1. The Study Population

**Figure 1:**
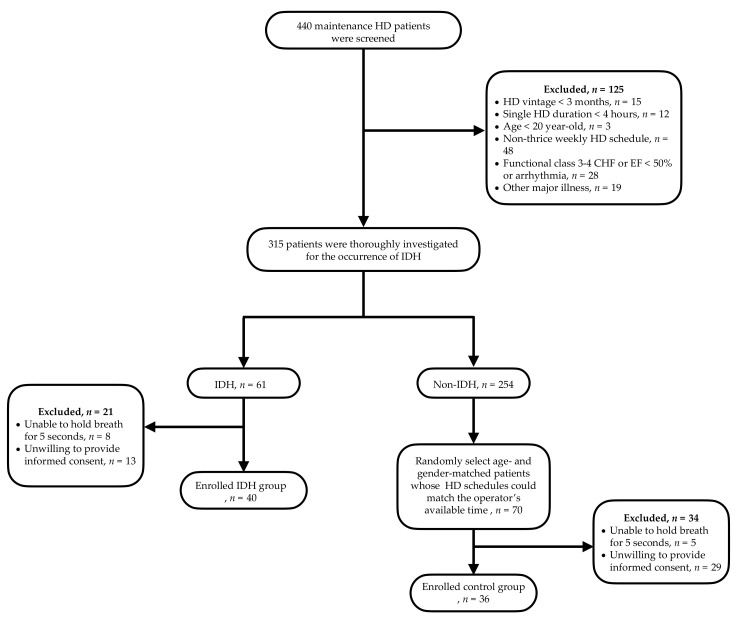
Flow chart of selection of study subjects.

Maintenance HD patients of the HD center of a university-affiliated hospital, the Chang Gung Memorial Hospital at Keelung, were enrolled. Patients aged greater than 20 years and undergoing 4 h HD therapy three times a week for at least 3 months were included for study. Overall, 440 HD patients were screened for the presence of IDH. There is no generally acknowledged definition of IDH. After referring to the Kidney Disease Outcomes Quality Initiative (KDOQI) and European Best Practice Guidelines [12], IDH was defined as having a drop of systolic blood pressure of >20 mmHg during dialysis, accompanied by any clinical events attributable to hypotension, such as dizziness, nausea, muscle cramp and other manifestations of tissue hypoperfusion, and in need of nursing interventions. Patients with presence of functional class 3–4 congestive heart failure or LV systolic function <50%, presence of cardiac arrhythmias (including atrial fibrillation), incapability of holding breath for 5 s during the performance of the echocardiographic examination, concurrent major illness (severe infection, malignancy or malnutrition) or unwilling to provide informed consent were excluded from study. Forty patients were assigned into the IDH group if the IDH episode had occurred in at least two-thirds of all the dialysis sessions for 3 months. Age- and gender-matched HD patients who did not develop IDH were enrolled into the normal control group (CON) (Figure 1). This study was conducted in adherence to the Declaration of Helsinki and was approved by the Ethics Committee of the Institutional Review Board at Chang Gung Memorial Hospital (IRB No. 94-1034B, 104-1159 and 202002535B0). Informed consent was obtained from all patients.

### 2.2. Transthoracic 2D Echocardiography Measurements

The echocardiographic studies were performed by a single experienced operator (NY Yang) using a transthoracic iE33 ultrasound system (Philips Medical Systems, Best, The Netherlands). The echocardiographic studies were conducted at a midweek HD session of a thrice weekly hemodialysis program (Wednesday or Thursday, depending on their HD schedule). All enrolled patients received three 2D echocardiography examinations in supine position during a single dialysis session: (i) pre-HD (at arrival to the dialysis center, before puncture of vascular access and connection with dialysis tubing); (ii) during HD (at two hours after initiating the dialysis session); (iii) post-HD (at the end of the HD session, when blood returning was completed). Apical four- and two-chamber views of the LV were also acquired with the patient in the left lateral decubitus position. Images were considered to be of good quality if the endocardium was visualized in all walls. Volumes using biplane Simpson’s rule were obtained from apical four- and two-chamber views. Measurements of echocardiogenic parameters, including M-mode interventricular septum to posterior wall ratio (mIVS/PW), M-mode left ventricular mass volume (mLVmass), M-mode left ventricular end systolic volume (mLVESV), ejection fraction (EF) calculated by Simpson’s formula, Tei index of myocardial performance, M-mode left ventricular end diastolic volume (mLVEDV), M-mode left atrium size (MLA size), E/A, DT and septal E/e’ were obtained using the software program installed on the ultrasound machine, with the EDV measured at the time of mitral valve closure and ESV measured on the image with the smallest LV cavity. The papillary muscles were included in the LV cavity. The inferior vena cava (IVC) was measured with patients in a supine position. A loop was obtained of the IVC in the M-mode at the IVC–right atrial junction after visualization of the IVC. The diameter of maximum and minimum IVC during respiration were measured. The inferior vena cava collapsibility index (IVCCI) was calculated using the standard formula ((IVC max − IVC min)/IVC max) × 100%.

### 2.3. Statistical Analysis

We performed a sample size calculation by G Power 3.1 software. Based on an effect size of 70%, a minimal of 70 total samples (35 per arm) was found to have a study power of 0.80 and alpha error probability of 0.05 under two-tail analysis. A study number of 76 patients was justified by the sample size calculation statement. Age and gender were matched to avoid possible confounding effects from baseline characteristics.

Categorical variables were presented as frequency and percentage and compared using chi-square test or Fisher’s exact test. Continuous variables were expressed as means ± standard deviation (SD) and compared using Student’s *t*-test or a Mann–Whitney U test. The Kolmogorov–Smirnov method was used to test normality of numerical variables. If the probability density function of a variable was not normal or Gaussian distributed, then data were log-transformed to approximate a normal distribution before analysis. Dynamic echocardiographic parameters (the baseline, during dialysis and post-dialysis values) were compared using a paired-difference *t*-test. A two-way repeated measures analysis of variance (ANOVA) was applied to detect any differences between related means over times between IDH and CON. A univariate, followed by multivariate, logistic regression analysis was applied to identify independent association between echocardiographic parameters and IDH, after adjusting for all potential confounders for IDH. All statistical tests were two-tailed and a *p*-value < 0.05 was considered statistically significant. Data were analyzed using SPSS 22.0 for Mac OS (SPSS Inc., Chicago, IL, USA).

## 3. Results

### 3.1. Subject Characteristics

After screening 440 stable HD patients, 76 (40, IDH; 36, CON) patients were recruited (Figure 1). The comparisons of demographics and clinical characteristics between enrolled and non-enrolled subjects are demonstrated in Table S1. The enrolled subjects were likely to have female predominance (63.2% vs. 47.1%, *p* = 0.009), younger age (62.41 ± 11.69 vs. 66.03 ± 13.28 year-old, *p* = 0.025), better hemoglobin (10.43 ± 1.07 vs. 6.62 ± 2.87 g/dL, *p* = 0.016), lower albumin (3.73 ± 0.68 vs. 3.99 ± 0.48 g/dL, *p* < 0.001), higher cholesterol (182.83 ± 38.41 vs. 153.76 ± 36.27 mg/dL, *p* < 0.001), higher Kt/V (1.84 ± 0.41 vs. 1.67 ± 0.33, *p* < 0.001), and lower cardiac/thoracic ratio (0.47 ± 0.05 vs. 0.52 ± 0.07, *p* < 0.001). The baseline demographic characteristics of patients are shown in Table 1. The IDH patients were more frequently to have diabetes (40% vs. 25%, *p* = 0.012) and to use glucose containing dialysate (85% vs. 44%, *p* < 0.001). No significant difference was found in anti-hypertension medications, ultrafiltration rate, Kt/V, interdialytic weight gain, dialysate temperature, low calcium dialysate, dialysis vintage and other co-morbidities. 

### 3.2. Hemodynamic Characteristics between Groups

The systolic blood pressures (SBP) during HD and post-HD were significantly lower in the IDH group (during HD, 98.9 ± 23.9 vs. 132.5 ± 31.2 and post-HD 120.1 ± 28.1 vs. 135.7 ± 24.8, *p* = 0.043, respectively). The diastolic blood pressure (pre-, during or post-HD) did not differ between the two groups (Table 2).

### 3.3. Dynamic Heart Function Assessments between Groups

The IDH patients had a higher during HD EF followed by a substantial decrease at the post-HD phase compared to the CON patients (change from pre-HD to during HD (△1): −2.29 ± 7.49 vs. 2.39 ± 7.46, *p* time * group = 0.024). The magnitude of changes of pre-during (△1) and during-post (△2) HD of mLVESV tended to be larger in IDH than that in CON patients (*p* time = 0.137), implicating more LV contraction of IDH patients during HD. However, the diastolic functions of IDH patients were impaired compared to that of the CON group. The mLVEDV and IVCCI of IDH patients were comparable to those of CON patients throughout the HD session (△mLVEDV, *p* time * group = 0.142; △IVCCI, *p* time * group = 0.64) (Table 3). The E/A ratio was lower in the IDH group than that in the CON group over time (pre-HD, 0.8 ± 0.2 vs. 1.0 ± 0.7; during HD, 0.7 ± 0.2 vs. 0.8 ± 0.3; and post-HD, 0.7 ± 0.3 vs. 0.8 ± 0.4, *p* time < 0.001, respectively). The septal E/e’ of the IDH group overtime was significantly higher than that of the CON group (20.5 ± 12.9 vs. 19.1 ± 0.6; 14.0 ± 5.7 vs. 16.9 ± 8.0; 18.0 ± 10.6 vs. 14.7 ± 6.7, *p* time < 0.001, respectively) and the fluctuations of septal E/e’ throughout the HD session were more prominent in the IDH than in the CON group (septal E/e’ △1 and △2, *p* time * group = 0.007) (Table 3). The DT of the IDH group was significantly longer than that of the CON group throughout the HD session (*p* time * group = 0.024), and the magnitudes of changes in DT of the IDH group were also greater than those of the CON group (*p* time * group = 0.038). The evolutions of parameters including MLA size, mIVS/PW, mLVmass and Tei index showed no significant difference between the two groups (Table 3).

### 3.4. Two-Way Repeated Measures ANOVA to Confirm the Differences of Cardiac Performance over Time between Groups

Significant echocardiographic factors identified in the former analyses were further explored in repeated measurement analyses to elucidate the differences in changes of cardiac performance between the IDH and CON groups over time. The changes in E/e’ over times were significant between the IDH and CON groups after adjusting for gender, age and ultrafiltration (*p* = 0.016, model 1) or adjusting for gender, age, ultrafiltration and diabetes (*p* = 0.076, model 2, Table 4).

### 3.5. Resampling Analysis Using a Subset of Patients with Diabetes Mellitus

Since the presence of diabetes mellitus was more prevalent among the IDH patients (Table 1) and in order to control the confounding effect of baseline characteristics, we resampled a subset of the diabetic patients. We further compared the cardiac performances of diabetic patients with or without IDH. Similar to the overall population, significant changes in LVESV, EF, E/A and E/e’ were found between diabetic IDH and diabetic CON patients. While elevations in EF were found in the during HD phase of IDH patients in the overall population, we observed a reduction of EF during dialysis of diabetic IDH patients compared to the diabetic CON group (*p* = 0.018). The directions of changes in other cardiac parameters (LVESV, E/A and E/e’) were similar in diabetic patients compared to the overall population. The reductions of E/e’ in the during HD phase of diabetic patients were greater in the diabetic IDH group than in the diabetic CON group (*p* = 0.026) as well as the decrease of the E/A in the during HD phase (Table 5). The findings implicated impairment of both systolic and diastolic functions in diabetic IDH patients, especially in the during HD phase.

### 3.6. Echocardiographic Predictors of IDH

Factors with significant mean differences between the two groups were used to estimate the risks associated with the occurrence of IDH (Table 6). A multivariate logistic regression analysis revealed an independent association of gender (adjusted odds ratio (OR) = 0.299; 95% confidential interval (CI) = 0.084–1.064; *p* = 0.062), ultrafiltration rate (OR = 1.621; 95% CI = 1.004–2.615; *p* = 0.048), diabetes (OR = 2.886; 95% CI = 0.945–8.817; *p* = 0.063), and septal E/e’ ratio (OR = 0.040; 95% CI = 0.003–0.606; *p* = 0.02) with the occurrence of IDH.

## 4. Discussion

The management of IDH in chronic HD patients remains a challenge for nephrologists. Heart failure is a grave and rising public problem worldwide and it is associated with repeated hospitalizations, reduced quality of life and high mortality rate [2]. While systolic dysfunction is often associated with significant morbidities [13,14], asymptomatic LV diastolic dysfunction with preserved EF (EF > 50%) can also associate with poor survival [15]. Dynamic changes of heart functions during the 4 h HD session have seldom been explored, especially in HD patient undergoing IDH. Here, we found that the increase of systolic function (EF) and reduction of diastolic function (E/e’) were greater in IDH patients than the CON group, especially during HD. However, both the systolic and diastolic dysfunction were present in diabetic IDH patients compared to their diabetic counterparts. The echocardiographic septal E/e’ was an independent predictor for the occurrence of IDH. Understanding dynamic transitions of cardiac performance during HD treatment can help to design appropriate therapeutic strategies to combat the IDH in HD patients.

Our findings of the association between diastolic dysfunction and IDH were similar to those of another study [16]. The mechanisms involving IDH are multifactorial, including inadequate intravascular volume refilling, autonomic dysfunction, abnormal vascular compliance and poor cardiac performance such as LV systolic and diastolic dysfunction and acute hypovolemia during ultrafiltration dialysis [17]. The LV hypertrophy (LVH) and lower LV compliance were common in HD patients experiencing hemodynamics instability during dialysis [18,19]. LVH is linked with systolic and, particularly, diastolic dysfunction in CKD patients and these heart dysfunctions were present in as high as 80% of patients at the time of dialysis initiation [20,21]. Our study has demonstrated that IDH patients had higher septal E/e’ and lower E/A at baseline and greater fluctuations in the during HD and post-HD phases, indicating the presence of significant LV diastolic dysfunction, than in the control group. The blood flows through the mitral valve during LV relaxation in diastole producing an early diastolic mitral velocity (E, LV inflow velocity pattern). Additional blood is then pumped through the valve during the late diastole phase with contraction of the left atrium (A). The E/A ratio can be altered in diastolic dysfunction, and the lower E/A indicates a worse diastole [22]. On the other hand, the early peak diastolic velocity of the mitral annulus (abbreviated as e’) is sensitive to LV relaxation and it is not easily affected by preload, as compared to the E. The e’ decreases in parallel with the deterioration of LV relaxation. Changes of the septal E/e’ ratio implicate impaired diastolic function and correlated with increased LV filling pressure and were found to be the major contributing factors to intradialytic hypotension. In this study, we found that the septal E/e’ ratio was an independent predictor for the occurrence of IDH, after adjusting for age, gender, ultrafiltration, diabetes and other echocardiographic parameters. The findings highlighted the importance of an assessment of diastolic function in all HD patients in managing their hemodynamic changes during a treatment session.

Several possible mechanisms contributing to diastolic dysfunction are common in HD patients. Hypertension and diabetes can lead to modifications of the proteins of cardiomyocyte reformation, such as myosin-binding protein C (CMyBP-C) [23] and sarcomere macromolecule [24], and consequently impair myofilament relaxation [25]. Cardiac oxidative stress can also contribute to diastolic dysfunction [26]. Uremia represents a status of oxidative stress for host homeostasis. Reactive oxygen species induce changes in Ca^2+^ handling proteins and increases of the Ca^2+^ sensitivity of myofilaments. The delay of Ca^2+^ extrusion from the cytoplasm, the increase of diastolic Ca^2+^ content and increase of myofilament Ca^2+^ sensitivity could result in diastolic dysfunction and heart stunning [27,28]. Increases of levels of transforming growth factor-β and connective tissue growth factor and alterations of degraded collagens during renal fibrosis have also been associated with diastolic dysfunction [29,30,31]. Our findings were consistent using repeated measurement analysis after adjusting for ultrafiltration rate and diabetes, highlighting an independent role of diastolic dysfunction on the pathophysiology of IDH of HD patients.

Our findings have also revealed that patients with IDH had greater increases in EF and greater decreases in mLVESV during HD. Previous studies have indicated a decline of EF at mid-HD as a possible predictor of IDH using real-time 3D echocardiography [8,16]. However, consecutive assessments of echocardiographic changes at three different time points of a single HD session enabled a more in-depth assessment of dynamic heart performances in IDH patients. In addition, the evaluation of systolic function not only relied on the EF but also on parameters of cardiac output, such as mLVEDV and decreased mLVESV. The ultrafiltration volumes did not differ between the two groups. In the presence of altered mLVEDV and decreased mLVESV of IDH patients, more hemodynamic monitoring should be needed to clarify the exact variation of real-time systolic function in IDH patients during a single HD session.

Consistent with many other studies, the prevalence of diabetes mellitus was higher in our IDH patients; however, decreases of both EF and E/e’ at the during HD phase were noted in our diabetic IDH patients. Diabetic end-stage kidney disease patients can coexist with vasculopathy and neuropathy. The micro and macroangiopathy can lead to atherosclerosis, arterial stiffness and myocardial abnormality, compromising systolic–diastolic performance and peripheral resistance [13,14]. Diabetic HD patients are more likely to have cardiac autonomic dysfunction causing sympathetic overactivity and vagal withdrawal associated with profound metabolic disarrangement, uremia and changes of heart geometry [32]. Such autonomic defects may cause myocardial infarction, malignant arrhythmia and sudden death [33]. Although the evaluation of autonomic dysfunction has not been conducted in the present study, the dynamic and comprehensive evaluation of heart performance may help to design strategies for optimizing systolic and diastolic functions to prevent the occurrence of IDH in diabetic HD patients.

Further work is needed to overcome a number of shortcomings of our study. First, we applied non-invasive methods to assess heart function and used the septal E/e’ ratio, decelerations time and E/A ratio as surrogate markers of LV diastolic function instead of direct hemodynamics monitoring. Our approach may misclassify the exact cardiac performance in condition of fluid overload; however, the testing on the midweek day of the HD program and at three specific time points (before, during and after) of a single HD session minimized the effects of volume and pressure overload on our estimates. The effect of septal E/e’ ratio remained a significant predictor of IDH with adjustment for ultrafiltration rate in both repeated measurement and logistic regression analysis. Second, autonomic dysfunction, intradialytic electrolyte alteration and coronary arteries flow reserve were not assessed. Common investigations of autonomic functions include heart rate response to deep breathing, beat-to-beat cardiac variation (R-R interval ratio) with changes of position or respiration, decline of blood pressure on standing for three minutes and others [34]. All these maneuvers were difficult to perform during a 4 h HD. Third, the effect of altered vascular component complicated by reduced vessel wall compliance and nonatherosclerotic arterial remodeling in HD patients was not assessed, which might contribute to IDH. Fourth, the enrolled subjects had several significantly different clinical characteristics in comparison to the non-enrolled ones, and the study results were only from a small part (17%) of our HD patients. Hence, the results probably cannot extend to our whole HD population. Fifth, at the time when the images were obtained, we did not have the ability to measure global longitudinal strain which may show the performance of the myocardium more directly, but we will certainly take this into consideration in future studies. Finally, the findings were derived from participants of a single center with unique ethnicity and a relatively small sample size. Although repeated measurements have strengthened the conjecture of our supposition, further large-scale studies are necessary to decipher the pathophysiologic and prognostic implication of IDH in dialysis patients.

## 5. Conclusions

We have demonstrated that the septal E/e’ ratio was a significant predictor for IDH and the dynamic LV diastolic dysfunction was the major contributing factor to the occurrence of IDH in patients on maintenance HD, independent of ultrafiltration rate and the presence of diabetes mellitus. In contrast, significant differences of both systolic and diastolic echocardiographic parameters over time were found in diabetic IDH compared to diabetic counterparts. However, the practical utility of the echocardiographic findings for a HD center practice is relatively limited, for it is difficult to perform routine echocardiography on IDH patients in current HD facility settings. At least, these findings provide one of the cardiogenic clues leading to IDH and remind us of early referral to cardiologists to jointly design therapeutic strategies focusing on cardiac dynamics, to prevent IDH in HD patients.

## Figures and Tables

**Table 1:** Baseline Characteristics of study population.

Characteristics	IDH (*n* = 40)	Controls (*n* = 36)	*p* Value
Age	62.53 ± 12.32	62.28 ± 11.14	0.927
Male Gender (%)	11 (27.5)	17 (47.2)	0.075
Body mass index	22.4 ± 3.3	22.7 ± 2.6	0.308
Comorbidities			
Diabetes mellitus (%)	23 (40)	9 (25)	0.012 *
Hypertension (%)	21 (52.5)	17 (47.2)	0.538
Heart failure (%)	5 (12.5)	4 (11.1)	0.564
Coronary artery disease (%)	5 (12.5%)	2 (5.7%)	0.438
Stroke (%)	3 (7.5)	3 (8.3)	0.561
Laboratory data			
Hemoglobin (g/dL)	10.6 ± 1.1	10.2 ± 1.1	0.136
Albumin (g/dL)	3.8 ± 0.3	3.7 ± 0.4	0.547
C-reactive protein (mg/L)	7.2 ± 12.0	24.0 ± 62.8	0.102
Calcium (mg/dL)	9.6 ± 0.9	9.5 ± 0.9	0.496
Phosphate (mg/dL)	5.0 ± 0.4	5.0 ± 0.4	0.978
i-PTH (pg/mL)	332 ± 351	267 ± 328	0.407
FGF23 (RU/mL)	50,743 ± 41,448	54,665 ± 40,721	0.825
Bilirubin (mg/dL)	0.620 ± 0.13	0.96 ± 1.71	0.215
Total cholesterol (mg/dL)	186 ± 36	178 ± 42	0.361
Medications			
ACEI	0 (%)	1 (2.9%)	1
ARB	4 (11.8%)	5 (17.2%)	0.721
Beta blockers	4 (14.8%)	7 (20.6%)	0.479
Diuretics	2 (6.9%)	5 (14.7%)	0.437
Dialysis parameters			
Dialysate temperature (oC)	35.8 ± 0.5	35.8 ± 0.5	0.916
Dialysate conductivity	141.0 ± 1.4	140.8 ± 1.4	0.542
Low calcium dialysate, *n* (%)	11 (27.5%)	9 (25%)	0.805
Glucose containing dialysate, *n* (%)	34 (85%)	16 (44%)	<0.001 *
Interdialytic weight gain (kg)	2.6 ± 1.2	2.1 ± 1.1	0.059
Dry weight (kg)	56.3 ± 12.9	57.3 ± 9.0	0.684
Ultrafiltration rate (mL/min)	11.2 ± 5.4	9.2 ± 3.8	0.074
Blood Flow (mL/min)	256 ± 39	267 ± 46	0.265
Kt/V	1.88 ± 0.46	1.79 ± 0.34	0.373
Dialysis vintage, month	76 ± 39	60 ± 35	0.058

Abbrevations: IDH, intradialytic hypotension; i-PTH, intact parathyroid hormone; FGF23, fibroblast growth factor-23; ACEI, angiotensin-converting enzyme inhibitor; ARB, angiotensin receptor blocker; Kt/V, mathematical formula representing a dose of dialysis. * Statistically significant, *p* < 0.05.

**Table 2:** Hemodynamic parameters during hemodialysis session between two groups.

	IDH (*n* = 40)	Controls (*n* = 36)	*p* (Time * Group)	*p* (Time)
Systolic pressure(mmHg)			0.043 *	0.041 *
Pre-HD	145.9 ± 30.8	145.2 ± 30.7		
During HD	98.9 ± 23.8	132.5 ± 31.2		
Post-HD	120.1 ± 28.1	135.7 ± 24.8		
Diastolic pressure(mmHg)			0.256	0.178
Pre-HD	73.6 ± 11.4	77.69 ± 16.3		
During HD	55.7 ± 11.0	68.3 ± 18.1		
Post-HD	67.7 ± 15.0	72.9 ± 12.0		

IDH: intradialytic hypotension, HD, hemodialysis. * *p* < 0.05.

**Table 3:** Echocardiographic parameters during hemodialysis session between two groups explored by two-way repeated measures ANOVA model.

	IDH (*n* = 40)	CON (*n* = 36)	*p* (Time * Group)	*p* (Time)		IDH (*n* = 40)	CON (*n* = 36)	*p* (Time * Group)	*p* (Time)
**Systolic parameters**									
mIVS/PW			0.571	0.514	△mIVS/PW			0.514	0.414
Pre-HD	1.34 ± 1.68	1.09 ± 0.33			△1	(−0.28) ± 1.72	0.06 ± 0.34		
During HD	1.06 ± 0.31	1.17 ± 0.30			△2	(−0.03) ± 0.37	(−0.07) ± 0.44		
Post-HD	1.03 ± 0.27	1.10 ± 0.34			△3	(−0.31) ± 1.69	0.01 ± 0.47		
mLVmass (g/m^2^)			0.466	0.42	△mIVS/PW			0.466	0.236
Pre-HD	201 ± 84	206 ± 80			△1	(−21.53) ± 69.23	(−6.11) ± 58		
During HD	180 ± 87	200 ± 84			△2	1.9 ± 55.32	1.49 ± 55.94		
Post-HD	183 ± 92	201 ± 84			△3	(−18.44) ± 62.32	(−2.31) ± 60.96		
mLVESV (mL)			0.657	0027 *	△mLVESV (mL)			0.554	0.137
Pre-HD	40 ± 20	35 ± 15			△1	(−4.25) ± 16.32	(−1.0) ± 14.82		
During HD	36 ± 24	34 ± 14			△2	5.05 ± 16.47	1.78 ± 17.62		
Post-HD	41 ± 29	36 ± 21			△3	0.79 ± 23.15	0.78 ± 16.59		
EF (%)			0.077	<0.001 *	△EF (%)			0.024 *	0.478
Pre-HD	58.1 ± 8.7	59.2 ± 7.8			△1	(−2.29) ± 7.49	2.39 ± 7.46		
During HD	77.1 ± 13.4	61.6 ± 7.0			△2	(−1.46) ± 8.92	(−1.02) ± 9.05		
Post-HD	54.6 ± 9.7	60.6 ± 9.8			△3	(−3.41) ± 8.34	1.29 ± 9.93		
Tei			0.507	0.08	△Tei			0.507	0.013 *
Pre-HD	0.28 ± 0.10	0.28 ± 0.10			△1	0.06 ± 0.17	0.05 ± 0.12		
During HD	0.33 ± 0.14	0.34 ± 0.10			△2	(−0.01) ± 0.14	0.02 ± 0.19		
Post-HD	0.32 ± 0.13	0.35 ± 0.20			△3	0.04 ± 0.14	0.08 ± 0.22		
**Diastolic parameters**									
MLA size (g/m^2^)			0.434	<0.001 *	△MLA size (g/m^2^)			0.434	<0.001 *
Pre-HD	38.2 ± 7.2	40.7 ± 6.9			△1	(−4.73) ± 5.6	(−3.83) ± 5.82		
During HD	33.5 ± 6.2	36.6 ± 7.9			△2	6.90 ± 32.19	0.88 ± 6.13		
Post-HD	40.2 ± 31.6	38.2 ± 8.0			△3	2.13 ± 32.63	(−2.66) ± 7.15		
Mitral E/A ratio			0.140	<0.001 *	△Mitral E/A ratio			0.14	<0.001 *
Pre-HD	0.8 ± 0.2	1.0 ± 0.7			△1	(−0.15) ± 0.24	(−0.27) ± 0.61		
During HD	0.7 ± 0.2	0.8 ± 0.3			△2	0.05 ± 0.25	0.02 ± 0.34		
Post-HD	0.7 ± 0.3	0.8 ± 0.4			△3	(−0.10) ± 0.29	(−0.25) ± 0.38		
mLVEDV (mL)			0.142	0.536	△mLVEDV (mL)			0.142	0.536
Pre-HD	101 ± 38	108 ± 36			△1	(−12.38) ± 32.72	10.00 ± 79.67		
During HD	89 ± 35	118 ± 76			△2	8.64 ± 28.21	(−14.64) ± 65.83		
Post-HD	98 ± 36	103 ± 37			△3	(−3.77) ± 37.62	(−4.64) ± 33.18		
DT (ms)			0.024 *	0.013 *	△DT (ms)			0.038 *	0.009 *
Pre-HD	240 ± 77	230 ± 75			△1	32.36 ± 91.48	28.26 ± 70.07		
During HD	271 ± 87	257 ± 80			△2	3.77 ± 85.85	(−42.2) ± 84.69		
Post-HD	275 ± 78	218 ± 84			△3	36.13 ± 96.73	(−14.97) ± 86.63		
Septal E/e’			0.007 *	<0.001 *	△Septal E/e’			0.007 *	<0.001 *
Pre-HD	20.5 ± 12.9	19.1 ± 0.6			△1	(−6.48) ± 10.91	(−3.26) ± 6.30		
During HD	14.0 ± 5.7	16.9 ± 8.0			△2	4.04 ± 8.67	(−2.21) ± 6.81		
Post-HD	18.0 ± 10.6	14.7 ± 6.7			△3	(−2.45) ± 7.93	(−4.44) ± 9.32		
IVCCI (%)			0.062	0.581	△ IVCCI (%)			0.067	0.64
Pre-HD	35.83 ± 19.14	40.57 ± 15.55			△1	5.89 ± 20.12	(−3.40) ± 18.10		
During HD	41.72 ± 19.10	37.17 ± 17.98			△2	3.30 ± 24.86	(−0.07) ± 21.32		
Post-HD	45.01 ± 18.66	37.11 ± 18.38			△3	9.19 ± 26.35	(−3.47) ± 22.73		

△1, during HD minus pre-HD; △2, post-HD minus during HD; △3, post-HD minus pre-HD; ANOVA, analysis of variance; IDH, intradialytic hypotension; CON, control group; HD, hemodialysis; mIVS/PW, M-mode interventricular septum to posterior wall ratio; mLVmass, M-mode left ventricular mass volume; mLVESV, M-mode left ventricular end systolic volume; EF, ejection fraction calculated by Simpson’s formula; Tei, Tei index of myocardial performance; mLVEDV, M-mode left ventricular end diastolic volume; MLA size, M-mode left atrium size; E/A, mitral inflow peak E to peak A velocity; DT, deceleration time; E/e’, septal ratios of transmitral flow velocity to annular velocity; IVCCI, inferior vena cava collapsibility index ratios. * *p* < 0.05.

**Table 4:** Two-way repeated measures ANOVA model to identify factors associated with IDH over time.

	*p* Value *	*p* Value **
mLVEDD (cm)	0.102	0.121
EF (%)	0.243	0.306
Deceleration time (ms)	0.096	0.155
Septal E/e’	0.016 ^$^	0.076

* Model adjusted for gender, age and ultrafiltration rate; ** model adjusted for gender, age, ultrafiltration rate and diabetes mellitus; ^$^ statistically significance, *p* < 0.05. mLVEDD: M-mode left ventricular end diastolic diameter; EF: ejection fraction; E/e’: septal ratios of transmitral flow velocity to annular velocity.

**Table 5:** Echocardiographic parameters in resampling subset of diabetes mellitus patients (*n* = 32).

	IDH (*n* = 23)	Controls (*n* = 9)	*p* (Time * Group)	*p* (Time)
Age, years	66 ± 12	65 ± 11		0.825
Ultrafiltration, kg	2.9 ± 1.4	2.2 ± 1.0		0.187
BMI	23.3 ± 3.7	23.2 ± 2.8		0.953
**Systolic parameters**				
LVEDV (mL)				
Pre-HD	99.1 ± 36	101.1 ± 31	0.154	0.327
During HD	91.3 ± 39	155.7 ± 14		
Post-HD	105 ± 38	115 ± 45		
LVESV (mL)				
Pre-HD	40.9 ± 20	38 ± 13.2	0.839	0.034 *
During HD	39.3 ± 28	35.2 ± 16.6		
Post-HD	47.9 ± 32.5	48.3 ± 29.7		
LVSV (mL)				
Pre-HD	58.2 ± 22.9	63.1 ± 22	0.156	0.292
During HD	52.0 ± 20.7	120 ± 14.7		
Post-HD	67.3 ± 19.7	67.3 ± 19.7		
EF (%)				
Pre-HD	56± 9	58 ± 7	0.174	0.018 *
During HD	54 ± 10	61 ± 6		
Post-HD	52 ± 11	54 ± 7		
**Diastolic parameters**				
Mitral E/A ratio			0.271	0.037 *
Pre-HD	0.76 ± 0.3	1.01 ± 0.4		
During HD	0.63 ± 0.2	0.91 ± 0.3		
Post-HD	0.67 ± 0.3	0.78 ± 0.3		
Deceleration time (ms)			0.06	0.385
Pre-HD	231 ± 83	235 ± 78		
During HD	254 ± 86	258 ± 11		
Post-HD	284 ± 81	204 ± 78		
Septal E/e’			0.075	0.026 *
Pre-HD	24.2 ± 15.7	25.3 ± 16		
During HD	15.1 ± 5.8	20.6 ± 9.9		
Post-HD	21.2 ± 12.8	16.4 ± 4.1		

BMI: body mass index; IDH: intradialytic hypotension; HD: hemodialysis; LVEDV: left ventricular end diastolic volume; LVESV: left ventricular end systolic volume; LVSV: left ventricular stroke volume; EF: ejection fraction by Simpson’s formula; E/A: mitral inflow peak E to peak A velocity ratios; E/e’: septal ratios of transmitral flow velocity to annular velocity fraction. * *p* < 0.05.

**Table 6:** Logistic regression analysis of factors associated with IDH.

Variables	Univariate Analysis		Multivariable Analysis	
	Crude OR (95% CI)	*p*	Adjusted OR (95% CI)	*p*
Age	1.002 (0.964–1.041)	0.926	-	
Male Gender (vs. Women)	0.424 (0.163–1.100)	0.078	0.299 (0.084–1.064)	0.062
Ultrafiltration rate (mL/min)	1.37 (1.046–1.796)	0.072	1.621 (1.004–2.615)	0.048 *
Diabetes (vs. no)	3.908 (1.462–10.452)	0.007	2.886 (0.945–8.817)	0.063
mLVESV (mL)	1.016 (0.990–1.043)	0.236	-	
EF (%)	0.984 (0.931–1.040)	0.560	-	
Tei	1.067 (0.009–1.235)	0.979	-	
mLVEDD (cm)	0.999 (0.938–1.063)	0.965	-	
Deceleration time (ms)	1.002 (0.996–1.008)	0.589	-	
Mitral E/A ratio	1 (0.952–1.051)	0.987	-	
Septal E/e’	0.171 (0.028–1.028)	0.054	0.040 (0.003–0.606)	0.02 *

Echocardiographic parameters of pre-HD phase were used for analysis. Backward stepwise selection method was applied for multivariate analysis. * *p* < 0.05. mLVESV: M-mode left ventricular end systolic volume; EF: ejection fraction calculated by Simpson’s formula; Tei: Tei index of myocardial performance; LVEDD: left ventricular end diastolic diameter; E/A: mitral inflow peak E to peak A velocity ratios; E/e’: septal ratios of transmitral flow velocity to annular velocity fraction.

## Data Availability

The primary data for this study are available from the authors on direct request.

## Supplementary Materials

The following are available online at https://www.mdpi.com/article/10.3390/diagnostics11122266/s1. Table S1. Demographics and clinical characteristics comparisons between enrolled subjects and non-enrolled subjects.
