# Supplementary material for: Dynamic Echocardiographic Assessments Reveal Septal E/e’ Ratio as Independent Predictor of Intradialytic Hypotension in Maintenance for Hemodialysis Patients with Preserved Ejection Fraction
Source: Diagnostics (Basel). 2021 Dec 3;11(12):2266. doi: 10.3390/diagnostics11122266 (PMC8700173; doi:10.3390/diagnostics11122266)
Supplement: Supplementary file 1 [file diagnostics-11-02266-s001.zip › diagnostics-1471820-supplementary.pdf]

Table S1. Demographics and clinical characteristics comparisons between enrolled and non-enrolled patients on hemodialysis

| | Enrolled (n= 200) | Non-enrolled (n= 298) | P value |
|---|---|---|---|
| Age, year | 65.08 ± 12.87 | 66.44 ± 13.55 | 0.264 |
| Male, n (%) | 101 (50.5) | 163 (54.7) | 0.357 |
| Anti-S1 Abs (EU/mL) | 2.33 (2.01-3.45) | N/A | N/A |
| Anti-RBD Abs (EU/mL) | 2.15 (1.94-2.92) | N/A | N/A |
| Hemoglobin (g/dL) | 10.06 ± 1.16 | 10.06 ± 1.31 | 0.993 |
| WBC (1000/μL) | 6.45 ± 2.74 | 6.73 ± 2.96 | 0.290 |
| Platelet (1000/μL) | 190.93 ± 67.60 | 190.01 ± 69.23 | 0.884 |
| Albumin (g/dL) | 4.04 ± 0.37 | 3.97 ± 0.55 | 0.149 |
| Cholesterol (mg/dL) | 154.83 ± 35.78 | 152.86 ± 36.66 | 0.562 |
| Triglyceride (mg/dL) | 115 (78.75-175.25) | 122 (81-184) | 0.540$ |
| AST (U/L) | 17 (13-21) | 17 (13-21) | 0.996$ |
| ALT (U/L) | 14 (10-19) | 15 (11-20) | 0.036*$ |
| Alk-P (U/L) | 92 (72.25-137.75) | 92 (71-136) | 0.732$ |
| Total bilirubin (mg/dL) | 0.4 (0.3-0.4) | 0.3 (0.3-0.4) | 0.398$ |
| Bun (mg/dL) | 69.93 ± 21.13 | 71.12 ± 22.03 | 0.548 |
| Creatinine (mg/dL) | 9.64 ± 2.50 | 9.22 ± 2.80 | 0.086 |
| Uric acid (mg/dL) | 6.22 ± 1.92 | 6.58 ± 1.73 | 0.032* |
| Na (meq/L) | 138.11 ± 3.15 | 138.07 ± 3.25 | 0.914 |
| K (meq/L) | 4.77 ± 0.79 | 4.65 ± 0.85 | 0.140 |
| Ca (mg/dL) | 9.40 ± 0.81 | 9.28 ± 0.94 | 0.160 |
| P (mg/dL) | 5.34 ± 1.57 | 5.21 ± 1.54 | 0.383 |
| C-reactive protein | 4.30 (1.40-9.42) | 3.35 (1.20-9.20) | 0.482$ |
| Urea reduction rate | 76 (71-80) | 76 (71-80) | 0.961$ |
| Kt/V (Daugirdes) | 1.65 ± 0.33 | 1.68 ± 0.32 | 0.421 |
| nPCR (g/kg/day) | 1.09 ± 0.49 | 1.07 ± 0.29 | 0.660 |
| TACurea | 41.48 ± 13.16 | 42.16 ± 13.46 | 0.586 |
| Iron (μg/dL) | 68 (51-90) | 65 (50.00-82.25) | 0.167$ |
| Ferritin (ng/mL) | 403.50 (223.00-654.75) | 437.00(173.00-675.25) | 0.860$ |
| TSAT (%) | 34.02 ± 13.89 | 32.23 ± 14.91 | 0.185 |
| Cardiothoracic ratio | 0.52 ± 0.06 | 0.52 ± 0.07 | 0.563 |
| Ca x P product | 50.32 ± 16.42 | 48.38 ± 15.09 | 0.176 |

Notes: Data are presented as mean ± standard deviation and median (interquartile range).
Abbreviations: Abs, antibodies; WBC, white blood cell count; AST, aspartate transaminase; ALT, alanine transaminase; Alk-P, alkaline phosphatase; Bun, blood urea nitrogen; Kt/V, A mathematical formula representing a dose of dialysis; nPCR, normalized protein catabolic rate; N/A, not applicable; TACurea, time average urea concentration; TSAT, transferrin saturation. *: statistically significant; $: Nonparametric, independent Sample Mann-Whitney U test.
